# Application of Gagné’s Instructional Design in Clinical Simulation: Enhancing Learning in Obstetric Emergency Management

**DOI:** 10.7759/cureus.76845

**Published:** 2025-01-03

**Authors:** Akshatha Daniel, Kavitha Nagandla, Shriyaa Daniel

**Affiliations:** 1 Emergency, West Middlesex University Hospital, London, GBR; 2 Obstetrics and Gynaecology, International Medical University (IMU) University, Kuala Lumpur, MYS; 3 Obstetrics and Gynaecology, University of Leeds, Leeds, GBR

**Keywords:** critical obstetric skills, gagné’s nine events, instructional design, postpartum hemorrhage, simulation-based learning

## Abstract

Introduction: Simulation‐based learning is a critical component in medical education, particularly for high‐risk scenarios like obstetric emergencies. This study utilized Gagné’s nine-step instructional model to design and evaluate a clinical simulation session on postpartum hemorrhage (PPH) management for fourth‐year medical students.

Methods: The session was structured using Gagné’s instructional events, including engaging case vignettes, multimedia presentations, guided practical activities, and immediate feedback. Pre‐ and post‐tests measured knowledge gains, while a 12‐item Likert scale questionnaire assessed student satisfaction. A focus group discussion (FGD) was conducted to explore student perceptions and analyzed thematically to identify key areas for improvement.

Results: The session demonstrated significant knowledge improvement, with post‐test scores (mean: 82.9%, 95% CI: 76.8-81.1) significantly higher than pre-test scores (mean: 68.4%, 95% CI: 60.7-66.1; p < 0.001). The instructional activities received high ratings (mean: 5/5), highlighting the alignment of objectives and content. The FGD thematic analysis revealed five key themes: "learning effectiveness", ʺrealism and applicationʺ, ʺfeedback and improvementʺ, ʺsession challengesʺ, and ʺrecommendations for enhancementʺ. Students suggested more diverse scenarios, extended debriefing sessions, and standardized feedback delivery.

Conclusion: The integration of Gagné’s instructional model into simulation‐based learning enhanced knowledge acquisition and student satisfaction. Recommendations for improvement include refining feedback mechanisms, increasing session diversity, and extending reflective discussions. This approach supports its utility in teaching critical obstetric skills and highlights opportunities for further enhancement.

## Introduction

In teaching and learning high-risk scenarios like obstetric emergencies, instructional design turns out to be a critical aspect in achieving the desired learning objectives. Simulation has gained popularity as an instructional method of medical education [1, 2]. The benefits of simulation-based learning are numerous and can stand undisputed [2]. It has been shown that simulation promotes skills acquisition by improving technical and psychomotor skills. Still, the greatest learning results can be seen in clinical decision-making and crisis resource management in health [3,4]. Obstetrics is a notoriously low-error-tolerant field. Research suggests that a high proportion of maternal deaths are preventable and are the result of human error [3]. It is incumbent to train healthcare professionals to enable them to transfer their skills into the clinical environment [5].

Instructional models play a vital role in developing teaching strategies and selecting the most effective method of designing instruction [6]. Gagné's instructional model, although behaviorist in nature, incorporates constructivist principles and is recognized for its focus on conditions of learning, making it highly impactful [7]. It is crucial to understand the differences between Gagné's model and other instructional theories in education, such as behaviorist and constructivist theories, as they each have specific instructional design sequences [8]. Gagné's model comprises nine events of learning, including gaining attention, informing the learner of the objectives, stimulating the recall of prerequisite learning, presenting the stimulus material, providing learner guidance, eliciting the performance, providing feedback, and assessing the performance, all of which are connected to inhibitory and excitatory pathways [9]. Such events of learning highlight how effective instruction aligns with the brain’s natural learning processes through the activation of excitatory and inhibitory neural pathways [9]. 

In the field of clinical simulation, Gagné's nine events of instruction can be applied to create scenarios and measure their impact on student learning outcomes [10]. The model has practical implications for curriculum assessment, constructive feedback strategies, and cognitive skill development for medical students [11]. Evaluating the impacts of the design of these simulations requires a combination of formative and summative evaluation [12]. It is essential to devote more interest and energy to the precise evaluation and publication of these new additions to the learning matrix for medical students [13].

The study aimed to assess the outcome of an instruction design based on Gagné's nine-step model of instruction delivery of a clinical obstetric emergency skills simulation session concerning the management of postpartum hemorrhage (PPH) to fourth-year undergraduate medical students on their obstetrics and gynecology rotation. This study seeks to present a detailed course plan, as well as to discuss the effectiveness of the instructional design.

This article has been previously posted to the Preprint server on December 9, 2024 (DOI: 10.20944/preprints202412.0649.v1).

## Materials and methods

The instructional design of the simulated session was prepared using a comprehensive lesson plan with Gagné's nine events of instruction in the context of PPH management. The nine events of instruction, including gaining attention, informing the learner of the objectives, stimulating recall of prerequisite learning, presenting the stimulus material, providing learning guidance, eliciting the performance, providing feedback about performance correctness, assessing the performance, and enhancing retention and transfer, were incorporated.

Gaining attention

The lesson plan started with a clinical case vignette, which was a real case presented in an emergency setting complicated by PPH. To focus students and stress the urgent need for timely action and intervention in PPH, analytical and thought-provoking questions like this were used: "*Imagine you are the first responder to a PPH emergency. What would capture your attention most in this scenario?*"

Informing the learners of the objectives

Explicit learning objectives were introduced at the beginning of each session, detailing the desired skills and knowledge that students were expected to obtain. An example of the objectives is as follows: "*Discuss the symptoms of PPH, first-line interventions, and applying clinical guidelines for management.*"

Stimulus (recall of prerequisite learning)

A short review of important topics, such as maternal physiology, labor and delivery process, and risk factors for PPH, was conducted to activate previous knowledge and assist students when learning new information. An example is as follows: "*Can you recall a time when you learned about the mechanisms of blood coagulation or uterine contraction? How do these mechanisms relate to managing PPH*?"

Present the stimulus material

Core content on PPH was delivered in a multimedia presentation, including causes, diagnosis, management protocols, and treatment algorithms as pre-learning materials in the e-portal. We supplemented it with real-life clinical scenarios for better contextual understanding. All the learning materials were available in the student learning management system (LMS). A sample is as follows: "*What are the key risk factors and potential complications associated with PPH that every healthcare provider should know?*"

Providing learning guidance

The simulation exercises were supported with gradual assistance. Instructors emphasized critical decision points and explained their reasoning for each intervention. For instance, the participants were asked, "*Given the symptoms and signs presented in this case, what guided questions would you ask to assess her condition more thoroughly?*"

Eliciting the performance

This was a hands-on practical activity with a high-fidelity manikin where students participated in simulated management of a PPH scenario. They had to complete activities including gauging the volume of blood loss, starting uterotonics, doing uterine massage, and escalating care as needed. An example of a question was, "*Based on our case study, what immediate actions would you take to manage Jane Doe's condition? List the steps in order of priority.*"

Feedback on the correctness of the performance

After each simulation, immediate feedback was provided on strengths and areas for improvement by the facilitator. Reactive feedback on clinical reasoning, procedural skills, and communication. The participants were asked, "*What do you think was the most effective part of your proposed management plan? What might you need to reconsider or adjust?*"

Assessing the performance

A knowledge test was given before and after the activity to evaluate the clinical understanding of PPH. Performance against clinical guidelines was also assessed using checklists for the performance of simulation modes. A sample question was, "*How would you modify your approach if the patient had a known allergy to commonly used medications in PPH management?*"

Improving retention and transfer

Learners were provided with summary handouts, practice scenarios, and access to online learning resources to facilitate learning. Facilitated discussion of real-world applicability of such knowledge-enhanced transferability. The participants were asked, "*How can you apply the skills and knowledge from this simulation to a real-world setting? What steps will you take to ensure you are prepared for such an emergency?*"

Evaluation of the instructional design model

The evaluation of the instructional design model was assessed using Kirkpatrick's evaluation framework with its focus on Levels 1 and 2, which looks at how satisfied students were and whether the learning objectives were achieved.

At the end of the session, a 12-item Likert scale questionnaire was administered to evaluate students' perceptions of this instructional design to evaluate student opinions and satisfaction. The items consisted of clarity of objectives, relevance of content, effectiveness of teaching methods, quality of feedback, and applicability to clinical practice. Responses varied from "strongly agree" to "strongly disagree."

The knowledge and skill acquisition was assessed using pre- and post-test 10 multiple-choice questions (MCQs) to assess students' understanding, clinical application, and critical thinking in PPH management. Pre-testing provided a baseline for knowledge, and post-testing provided an analysis of learning gains. The improvement was analyzed with paired comparisons. A checklist of 10 key simulation actions, such as identifying PPH, treating the PPH, and escalating care, was used to assess performance during the simulation (Appendix A).

To further determine students' perceptions, a focus group discussion was conducted using qualitative thematic analysis to generate key themes on recommendations.

Data collection and analysis

Quantitative Data

Statistical analysis of the pre-and post-test knowledge scores was performed using paired t-tests to evaluate learning gains.

Students’ perceptions of the lesson design were assessed using five-point Likert-scale responses, which were analyzed to derive mean scores and trends (Appendix B).

Qualitative Data

The qualitative data collected through open-ended questions from students provided details about students' experience gaining new insights into their learning and academic development, as well as where they felt there could be improvement.

The focus group discussion involved 10 students, and the discussion involved the following open-ended questions:

a. What role did the clinical simulation sessions play in your learning and confidence? b. What were the key learnings from the sessions? c. What did you have to overcome in the simulation? d. What can be done to improve the sessions (setup, feedback, realism, etc.)?

The thematic analysis of the focus group discussion (FGD) on learning of clinical simulation using instructional design was conducted in the following manner. First, the transcripts from the discussion were read through to better understand how the students felt about the sessions. Coding was first conducted inductively by identifying excerpts on participants' experiences, barriers, and recommendations. These codes were organized into broader categories, from which key themes emerged, such as “learning effectiveness”, "realism and application”, “feedback and improvement”, “session challenges”, and “recommendations for improvement”. The themes were checked for consistency and confirmed through quotes from the discussions to reflect the views of participants. The final report generated a thematic map along with evidence providing actionable guidance on how to enhance the design and delivery of simulation-based learning by applying instructional design.

## Results

The simulation-based teaching session for PPH was designed using Gagné's nine-step instructional model and was administered to 94 fourth-year medical students. The session was assessed with student feedback, pre- and post-assessment of basic knowledge, and free comments for further improvements. Below is an elaborate evaluation of the results.

Overall perception of instructional activities and learning goals

Students responded with an average of five (on a Likert scale) for both the instructional activities, learning objectives, and the content. This reflects a good alignment between the pedagogical design and student expectations for clear objectives, relevant subject matter, and engaging activities. This high rating indicates that Gagné's instructional design provided a systematic method that supported the learning.

Assessment, feedback, and usability

The overall usability rating of the e-portal and feedback mechanics embedded in the instructional design was 4.2 ± 0.5. In comparison, this lower score identifies a need for further refinement of feedback and assessment processes. The e-portal is user-friendly, but qualitative comments indicated that there is a need for improvements in the depth and quality of feedback.

Knowledge improvement

Scores on Pre- and Post-training Tests

The knowledge scores increased significantly following the training session. The pre-training test score was 68.4% (95% CI 60.7-66.1) and the post-training test score was 82.9% (95% CI 76.8-81.1). The difference in scores pre- and post-training was statistically significant (P <. .001), which demonstrates the impact of the instructional design and simulation-based intervention in enhancing competency acquisition. The confidence intervals imply a uniform improvement across the student cohort, confirming the mechanism's validity. The results are presented in Table 1.

**Table 1:** Pre- and post-test scores (Mean, SD, t scores, and p-value) PPH: postpartum hemorrhage

Scores	Pre-test scores (n=94)		Post-test scores (n=91)				t-test
	Mean	SD	Mean	SD	Value	
PPH multiple choice question	68.4 (95% CI 60.7-66.1)	5.5	82.9 (95% CI 76.8-81.1)	7.3	−10.8*	
*P < 0.001						

Suggestions for improvement

Below is the qualitative feedback from open-ended comments toward improving future efforts in instructional design.

Training Session Repetition

Students expressed the need for additional simulation sessions during the obstetrics and gynecology rotation. Repetition was proposed to build knowledge retention and strengthen skills through practice over time.

Increase Time for Debriefing

Having more time for debriefing was suggested. This would enable further discussions of the simulation, detailed error reviews, and clarification of key concepts.

Formative Assessments with Detailed Feedback

Students requested detailed feedback on formative questions. This information would give them more distinct data about their performance that could be used to focus on enhancing their clinical reasoning and decision-making.

Based on the General Framework for Qualitative Content Analysis proposed by Braun and Clarke, five main themes and their corresponding subcategories were identified from the focus group discussion with students regarding the clinical simulation learning experience during the fourth year [14]. Findings from the thematic analysis by FGD on clinical simulation learning are presented in Table 2.

**Table 2:** Thematic analysis of the focus group discussion

Key Themes	Sub-themes	Findings	Verbatim
Theme 1: The effectiveness of learning	Enhanced practical skills improved clinical reasoning	Students indicated increased confidence in performing clinical skills, such as uterine massage or initiating uterotonics due to simulation. In critical situations, scenarios helped students to put theory into practice.	Quote: “Doing it hands-on helped me remember what to do when you were in a real-life emergency.”; Quote: “The simulation pushed us to think on our toes and prioritize tasks.”
Theme 2: Realism and applicability	Authenticity of scenarios: lack of diversity in cases	Students felt that scenarios closely resembled real clinical cases, which also enhanced their preparation. Some students said scenarios focused on fairly common conditions but didn’t include rarer, high-risk cases.	Quote: “The setup was like a real labor ward, so it felt more natural and easier to get into the scenario.”; Quote: “To include complex cases such as eclampsia or retained placenta would be great.”
Theme 3: Feedback to change	Detailed feedback: not delivering consistent feedback:	Praise was given for constructive and specific feedback on clinical performance, but students requested more specific information about decision-making errors. Some had heard of significant quality and depth differences in feedback among different facilitators.	Quote: “Knowing what we did right and wrong so soon after helped us improve.”; Quote: “Feedback provided was not always consistent.”
Theme 4: Session challenges	Time constraints	Some of the students said they felt pressured for time during some of the simulations, which prevented them from fully applying themselves to the scenarios.	Quote: “We needed more time to think and to act, especially in the debriefing.”
Problems related to resources and technology	Equipment malfunction	There were no specific issues pointed out by the students.	Quote: “The manikin functioned well and some minor issues were tackled by the facilitators."
Theme 5: Suggestions for enhancing	Add in more complicated scenarios: extended debriefing sessions	Students recommended new advanced, cross-cutting cases incorporating obstetric emergencies and neonatal resuscitation. Students highlighted the desire for more time to talk about mistakes, how they could do things differently, and how they did overall.	Quote: "It would be “very useful” to work with a pediatric team in a simulation."; Quote: “We need more time to understand what went wrong and how to do better.”

## Discussion

The result of this study validates the effectiveness of the instructional design model within simulation-based learning in the context of obstetric emergencies in undergraduate settings. One of the valuable tools in preparing future graduates is procedural and clinical skills to apply in clinical settings. Such hands-on skills in high-risk scenarios, such as obstetric emergencies, practice and apply psychomotor skills in controlled settings, ensuring competency in real settings [4,15]. Gagné's model of instructional design was perceived positively by the students related to its structure and relevance as it organized the learning process in achieving the learning objectives. The cognitive theories ensure learning are actively engaged, receive guidance, and apply new knowledge effectively. Such instructional design models embedded with cognitive theories are expected to have a positive learning experience with knowledge retention and clinical application as opposed to traditional teaching methods [16]. The results of pre-and post-training knowledge assessment showed a statistically significant improvement in scores, from 68.4% to 82.9% (P < .001), which concurs with prior research on simulation-based training, along with structured instructional design, as an effective instruction design in medical education, especially in the context of critical skills such as obstetric emergencies [2].

An increase in overall student satisfaction ratings of an average of 5 underscores the effectiveness of such a design in achieving the learning objectives. Such findings are consistent with a study by Perrotta et al. that highlighted the need for such a structured approach in simulation learning. It is expected that such elements in the delivery of simulation learning foster better engagement and motivation among learners.

On the feedback component following the session, there was a slightly diminished score of 4.2 ± 0.5, which highlights an area for improvement. Studies have shown that detailed feedback helps learners better understand their strengths and weaknesses, which is critical for developing clinical reasoning and decision-making skills [17].

While students appreciated the feedback following the simulation during debriefing, qualitative remarks suggested that students anticipated more detailed feedback to enhance their learning. The cornerstone of medical education is providing effective feedback to the learners with actionable strategies for future improvement [18]. Debriefing is an important component of simulation-based learning, which allows learners to reflect on their performances, as it provides an opportunity for learners to reflect on their performance and future strategies for improvement [19]. This study highlighted the likely benefits of debriefing sessions, and future iterations of the session will allow extended time for these sessions, allowing greater depth of discussion and reflection. Further, strategies to enhance feedback to be considered include peer feedback and rubric-based assessment to enhance the effectiveness of the instructional design.

The results of the FGD concur that Gagné’s nine events of instruction were effectively applied in the clinical simulation sessions to facilitate student learning. Case vignettes that felt realistic held the learners' attention, and didactic learning objectives gave clarity and focus that were in line with Gagné’s emphasis on motivation and goal-setting. Active student engagement in hands-on simulation activities strengthened procedural and clinical reasoning skills [20].

In essence, immediate feedback, although at times slightly inconsistent, was a significant aspect in identifying strengths and areas of improvement [21]. Tests (pre- and post-) confirmed significant knowledge gains, validating the merit of the instructional design [22]. Supplementary materials reinforced retention and enabled participants to apply their learning beyond the sessions. Recommendations on increasing learning outcomes also include better standardizing feedback, using more diverse scenarios, and optimizing debriefing sessions [19]. Gagné’s model overall afforded structured and impactful delivery of the simulation-based education.

The findings of this study have important considerations in medical education in developing teaching and learning strategies with the integration of Gagné's nine-step model as part of instructional design for an effective learning experience for undergraduate medical students. Evaluation results highlight the need to refine instructional designs by enhancing qualitative feedback, extending debriefing time, and repeating the sessions to facilitate reinforcement.

Limitations and future directions

There are several limitations to this study. While the results of this study are encouraging, several limitations should be considered. First, the study focused on a single cohort of fourth-year medical students, which may limit the generalizability of the findings. Future research could explore the applicability of this instructional design to other healthcare professionals, such as residents or nurses, and in different clinical contexts as interprofessional collaborative learning. 

Second, the evaluation of learning outcomes was limited to knowledge improvement and student satisfaction. Future studies should consider incorporating more objective measures of skill performance, such as video analysis of simulation sessions or real-world clinical outcomes.

Finally, the long-term impact of the instructional design on knowledge retention and clinical performance was not assessed. Longitudinal studies could provide valuable insights into the sustained benefits of integrating Gagné’s instructional model with simulation-based training.

## Conclusions

The integration of Gagné’s nine-step instructional model with simulation-based learning proved to be an effective approach for teaching the management of postpartum hemorrhage to medical students. The significant improvement in knowledge scores and high student satisfaction ratings underscore the value of this instructional design in enhancing learning outcomes. However, feedback from students highlights the need for further refinements, including repeated training sessions, extended debriefing time, and more detailed feedback mechanisms.

By addressing these areas for improvement, educators can optimize the instructional design to better meet the needs of learners and ensure that they are well-prepared to manage obstetric emergencies. These findings contribute to the growing body of evidence supporting the use of structured instructional models and simulation-based learning in medical education, ultimately leading to better patient care and outcomes.

## Appendices

Appendix A

Postpartum Hemorrhage Simulation (Pre- and Post-test Questionnaire)

**Table 3:** Postpartum hemorrhage simulation (pre- and post-test multiple choice questions)

Item no.	Postpartum hemorrhage simulation (pre- and post-test multiple choice questions)
1.	An 18-year-old primigravida delivered 30 minutes ago continues to have trickling of blood from the vagina. The uterus is not well contracted. The placenta is completely expelled. There is no genital trauma. Her BP and pulse are normal. What is the most appropriate treatment? A. Oxytocin (Syntocinon) 10 IU intramuscularly B. Oxytocin (Syntocinon) 5 IU intravenous injection C. Oxytocin (Syntocinon) infusion D. Oxytocin/ergometrine (Syntometrine) intramuscular injection
2.	You are called to see a 34-year-old G2 P1 at 32 weeks, who is brought to A&E by her husband who found her unconscious at home. What is the initial assessment of this patient? A. Plan for immediate surgery B. Check for response and pulse C. Immediate ultrasound D. Intravenous fluid resuscitation
3.	A 35-year-old G8 P7 in early labor is admitted to the labor room. While examining her you notice she is tachypnoeic with a respiratory rate of 30/min. BP 100/40mmHg, SpO2 85% on air. What would be the most appropriate action? A. 15/liters oxygen per minute O2 via Non- rebreather mask B. Call an anesthetist and intubate patient C. Check the fetal heart sounds D. Bolus intravenous 0.9% normal saline
4.	36- year -old, G2P1 at 24 weeks gestation with gestational diabetes is admitted to the ante-natal ward for observation and control of her blood sugar. On admission, her blood pressure is 140/85 mmHg and her urine is (2+) for proteins. During the evening round, the nurse informs you that the patient is complaining of epigastric pain. While you are examining her, she starts to have tonic-clonic seizures that abort spontaneously, and she becomes mildly confused. What will be the appropriate management of this patient? A. Perform primary survey B. Intravenous fluid bolus of N/S C. Place in left lateral position D. Start oxygen therapy
5.	A 27-year-old gravida 3 para 2 at 41 weeks of pregnancy is admitted to active labor. Her pregnancy is low risk. She has well-controlled bronchial asthma. She delivers vaginally following a prolonged labor. Shortly afterwards she begins to have heavy bleeding with blood clots. Her blood pressure is 130/76 mm Hg, PR is 90 bpm, RR is 17/min. Examination reveals a ‘soft boggy uterus’. She is given bolus doses of oxytocin IV. Laboratory results show Hb 10.8g/dl, hematocrit 32.2 %, platelet count of 140,000 cells/mm3, prothrombin time of 14 secs, and partial thromboplastin time of 38 secs. Despite these measures, bleeding continues. What is the next step in the management? A. Blood transfusion B. Balloon tamponade C. Carboprost IM D. Tranexamic acid IV
6.	Which of the following is the correct dose of drug prescription in the management of postpartum hemorrhage for uterine atony? A. Carboprost 250 mg Intramuscular B. Oxytocin 5 IU IV / Ergometrine 500 microgram Intramuscular C. Misoprostolol 200 microgram PR D. Oxytocin 40 U in 500 ml N/S infusion
7.	A 44- year- old G4 P3 A3 has just had a spontaneous vaginal delivery after prolonged labor. The uterus is not well contracted but it improves after uterine massage However, she continues to bleed per vaginally. Her blood pressure drops from 110/70 to 90.50 mm Hg. Her pulse rate is 125/min. She is beginning to become restless. What would be the fluid of choice for this patient? A. Gelafundin B. Blood transfusion C. 0.45% saline D. Hartmann’s solution
8.	One hour after a spontaneous vaginal delivery, A 25- year- old primigravida is noted to be drowsy and confused. Her blood pressure is 92/55mmHg and PR is 122/min. You notice that she is clinically pale. The sanitary pad is fully soaked with blood clots on the bedsheets. There is active oozing of blood from the vagina. What will be your immediate action? A. Insert two 16G needles with IV crystalloids bolus B. Immediately perform a speculum examination C. Initiate - RED ALERT D. Oxygen therapy with a non-rebreather mask
9.	Thirty minutes after delivering her second child, a 32-year-old woman experiences sudden shivering and a rapid drop in blood pressure to 85/50 mmHg. She appears disoriented and her skin is cool and clammy. The medical staff notices a heavy flow of blood with some clots on the delivery bed. Her pulse rate is 110 beats per minute. What is the most appropriate immediate action? A. Administer an IV infusion of warmed crystalloids. B. Initiate a rapid pelvic ultrasound. C. Activate the emergency hemorrhage protocol. D. Give antibiotics and start temperature monitoring.
10.	A low-risk woman in her first pregnancy has an uncomplicated labor and has spontaneous vaginal delivery. Which of the following is the most appropriate uterotonic agent of choice for the management of 3rd stage of labor? A. Oxytocin (Syntocinon) 10 IU intramuscularly B. Oxytocin (Syntocinon) 5 IU intravenous injection C. Oxytocin (Syntocinon) 10 IU intravenous injection D. Oxytocin/ergometrine (Syntometrine) intramuscular injection

Appendix B

Likert Scale Questionnaire 

**Table 4:** A 12-item Likert scale questionnaire

Category /Items		Likert Scale				
Instruction Activities, Learning Objectives, Content		1 Strongly Disagree	2 Disagree	3 Neutral	4 Agree	5 Strongly Agree
1	The learning objectives were clearly defined at the beginning of the course.					
2	The content delivered was relevant to the learning objectives.					
3	The instructional activities were engaging and interactive.					
4	The pacing of the content was appropriate for the course duration.					
5	The instructional methods enhanced my understanding of the topic.					
6	The course provided practical examples and applications of the content.					
Overall Usability Rating of the E-Portal						
7	The e-portal was easy to navigate.					
8	The e-portal’s design was visually appealing.					
9	I could find all necessary resources and links without difficulty.					
10	The e-portal was consistently available with no technical problems.					
11	The assessment tools on the e-portal provided meaningful feedback.					
12	The feedback from assessments was timely and helped improve my learning experience.					
	Open comments if any					
